# Morning Cortisol Levels and Perceived Stress in Irregular Shift Workers Compared with Regular Daytime Workers

**DOI:** 10.1155/2012/789274

**Published:** 2012-06-18

**Authors:** Harri Lindholm, Jari Ahlberg, Juha Sinisalo, Christer Hublin, Ari Hirvonen, Markku Partinen, Seppo Sarna, Aslak Savolainen

**Affiliations:** ^1^Centre of Excellence of Health and Work Ability, Finnish Institute of Occupational Health, 00250 Helsinki, Finland; ^2^Institute of Dentistry, University of Helsinki, 00014 Helsinki, Finland; ^3^Department of Cardiology, Helsinki University Central Hospital, 00029 Helsinki, Finland; ^4^Centre of Excellence of Human Factors at Work, Finnish Institute of Occupational Health, 00250 Helsinki, Finland; ^5^Helsinki Sleep Clinic, Vitalmed Research Centre, 00420 Helsinki, Finland; ^6^Department of Public Health, University of Helsinki, 00014 Helsinki Helsinki, Finland; ^7^Finnish Broadcasting Company and Department of Public Health, University of Helsinki, 00014 Helsinki, Finland

## Abstract

The 24/7 work environment and irregular shifts may markedly enhance the psychological pressure of media work. Changes in the hypothalamic-pituitary-adrenal axis reflect adaptation to stress. We analysed the correlation between subjective stress, sleep, salivary cortisol, and melatonin hormones among Finnish media workers with regular daytime work (RDW) and with irregular shift work (ISW) while controlling confounders. From 874 employees with regular daytime work or with irregular shift work, 70 employees from both groups were randomly selected. The final number of employees with a complete salivary cortisol profile was 66 in the RDW group and 65 in the ISW group. Five saliva samples were gathered from each subject before and during a working day. The salivary cortisol level of the sample taken 60 minutes after awakening (T1) was compared to the salivary cortisol level taken immediately after awakening (T0, T1/T0 ratio). The ratio was higher in the ISW group than in RDW group. Irregular shift work (*P* < 0.001), severe stress (*P* < 0.05), and less sleep (*P* < 0.05) were independently associated with an augmented cortisol response after awakening. A stressful work environment and irregular shift work enhance cortisol excretion after waking. In the long run, this may become detrimental to health.

## 1. Introduction

The hypothalamic-pituitary-adrenal (HPA) axis is one of the main components of the stress adaptation system in humans [1]. Bursts of cortisol excretion oscillate diurnally and the amplitude of these bursts increase during morning hours. Environmental factors and mental stress may disrupt the balance in this cycle [2]. Augmented cortisol responses have been associated with metabolic syndrome, atherosclerosis, osteoporosis, immunosuppression, and an increased risk of coronary heart disease [1, 3]. Work-related stressors have been associated with augmented cortisol responses [4]. Poor recovery, long working hours, and an extensive physical workload affect cortisol reactivity [5–7]. Also short sleep duration and self-reported sleep disturbances may disrupt diurnal cortisol secretion patterns [8].

Combined evaluation of subjective and physiological stress might be useful to detect risk groups and determinants of poor health outcomes from working life. Ideally, the tools should be easily implemented in occupational health care. According to Elo and collaborators [9], a single question can be used in the evaluation of job stress. Salivary cortisol analyses have been used to monitor the HPA axis function [10]. In addition to self-reported sleep, wrist actigraphy can be used to reveal information about sleep duration [11].

Early morning responses in salivary cortisol excretion profiles have been used in the assessment of stress-related adaptation [12–14]. An enhanced cortisol awakening response (CAR) is associated with an increased level of stress [15]. Controlled timing for the salivary stress biomarkers has been recommended, and the importance of the cortisol response during the first 30 minutes after awakening has been underlined [16, 17]. In some studies, a sampling period of 60 minutes after awakening has also been used [18]. There are controversial results of the influence of the waking time on CAR. The majority of the studies reveal that early morning awakening causes a larger CAR [19].

Salivary melatonin (Sa-Mel) may serve as an indicator of a biomarker of circadian dysregulation [20]. The salivary cortisol (Sa-Cor) reflects the free plasma cortisol [21]. In the highly controlled environment, the cortisol concentrations increase from 50% to 160% in the first 30 minutes after awakening [22]. In real-life settings, there are individual differences in the waking-up period. The timing of the sample collection and, for example, snoozing might decrease the CAR. In the field studies, even negative responses have been reported in about 25% of the study subjects [23]. The dynamic nature of cortisol secretion enables the analyses of other indicators of stress responsiveness throughout the day. In all cases, the sampling protocol must be carefully planned [24].

Technological changes in the media industry call for new professions and competence requirements, whereas some existing skills are becoming redundant. The 24-hour culture of modern media work, with its irregular shifts and night-work, enhances the psychological pressures of work in demanding work environments. In one study, every fourth media worker reported having much or very much stress [25].

The aim of the present study was to analyse the association between physiologically measured and subjectively reported stress in Finnish media workers with irregular shift work or regular daytime work.

## 2. Materials and Methods

A standardized questionnaire was mailed to all employees of the Finnish Broadcasting Company with irregular shift work (ISW, *n* = 750, 57% men) and to an equal number of randomly selected controls in the same company with regular, eight-hour daytime work (RDW, 42% men). The questionnaire covered demographic items, employment details, general health, physical status, insomnia symptoms, psychosocial status, stress, work satisfaction, and performance. The work duties of the media personnel included journalism, broadcasting, programme production, technical support, and administration. The overall response rate was 58% (54% men). The response rate in the ISW group was 82% (54% men) and in the RDW group 34% (47% men).

Of the survey respondents (*n* = 874), 70 employees from both groups were randomly selected to participate in physiological measurements, including Sa-Cor and Sa-Mel profiles. The study subjects were asked to give their salivary samples in five numbered tubes on the preceding evening at bedtime, immediately after awakening, and one, three, and eight hours after awakening. The subjects were also asked to write, after giving the salivary sample, the exact sampling time onto the label of each tube. After collections of the samples, the tubes were transported to the nearby laboratory at the Finnish Institute of Occupational Health. The awakening time was between 3 AM and 5 AM in 75% of the ISW workers, between 5 AM and 7 AM in 20% of the workers, and later than 7 AM in 5% of the workers. The awakening time was between 5 AM and 7 AM in 70% of the RDW workers, between 7 AM and 8 AM in 20% of the workers, and between 8 AM and 9 AM in 10% of the workers.

The cortisol responses after awakening were calculated as the difference between absolute Sa-Cor levels in samples taken immediately (T0) and 60 minutes after awakening (T1), and as a ratio between the T1 and T0 sample.

The final number of employees with a complete Sa-Cor profile was 66 in the RDW group and 65 in the ISW group. Melatonin levels were available from 64 subjects in the RDW group and 65 subjects in the ISW group. The general health habits and psychosocial factors among the workers with ISW or RDW have been reported in our earlier study, and they did not differ significantly [26].

Sa-Mel levels were measured using a commercially available Direct Saliva ELISA kit (Bühlmann Laboratories AG, Schönenbuch, Switzerland). The assay is based on the competition principle and microtitre plate separation. Briefly, an unknown amount of melatonin present in the sample and a fixed amount of biotinylated melatonin compete for the binding sites of antibodies coated onto wells. After three hour's incubation, the wells are washed to stop the competition reaction and the enzyme label, streptavidin-conjugated to horseradish peroxidase (HRP), is added. During one-hour incubation, this binds to the melatonin-biotin-antibody complex captured on the coated wells. The unbound enzyme label is then removed by a second washing step, and a TMB (tetramethylbenzidine) substrate is added to the wells. In a third half-hour incubation step, a coloured product is formed in inverse proportion to the amount of melatonin present in the sample. The colour turns from blue to yellow after the addition of an acidic stop solution and can be measured at 450 nm. Due to the diurnal fluctuation of melatonin levels in humans, five samples were collected from each subject during the working day.

The Sa-Cor levels were measured using a commercially available luminescence immunoassay for the quantitative determination of cortisol in human saliva (Cortisol Saliva LIA, IBL Immuno-Biological Laboratories, Hamburg, Germany). The assay is based on the competition principle and microtitre plate separation. Briefly, an unknown amount of cortisol present in the sample and a fixed amount of Horseradish-Peroxidase-(HRP-) conjugated cortisol compete for the binding sites of the antibodies coated onto the wells. After three hour's incubation, the wells are washed to stop the competition reaction. After three hour's incubation, the wells are washed to stop the competition reaction and the luminescence solution is added. The relative luminescence units (RLUs) can be read after 10 minutes, and within 40 minutes. The concentration of cortisol is inversely proportional to the luminescence measured.

Actigraphy (Cambridge Neurotechnology Ltd., UK) was used to evaluate sleep/wake periods of the workers [27]. One minute epochs were used in the analysis. The awakening times were classified by one hour epochs.

Stress experience was assessed using the validated single-item measure of the Occupational Stress Questionnaire [9] as follows: “Stress means a situation when a person feels tense, restless, nervous or anxious, or is unable to sleep because his/her mind is troubled all the time. Do you feel that kind of stress these days?” (1 = “not at all,” 2 = “only a little,” 3 = “to some extent,” 4 = “rather much,” and 5 = “very much”).

All subjects gave their written informed consent. Study protocols were approved by the Ethics Committees of the Hospital District of Helsinki and Uusimaa, Helsinki, Finland.

Repeated measures of variance with the Huynh-Feldt within-subject effect test and including awakening time as a covariate in the models were used to estimate whether the variance of Sa-Cor and melatonin scores was dependent on the starting hour of collection. It turned out that the Sa-Cor profiles were not dependent on the awakening hour. In contrast, the melatonin profiles were highly dependent on the awakening hour, and thus only bedtime melatonin levels were used in the analyses for the present study.

A logarithmic transformation (ln) for the Sa-Cor T1/T0 ratio was computed due to the skewness of the distribution. This was also used as the dependent variable in a multiple linear regression model. Independent variables entered in the model were shift work (no = 0, yes = 1), severe stress (“very much”) (no = 0, yes = 1), and actual sleep time during the preceding night (hours). The model was adjusted for age and gender. Due to the asymmetry of the distribution, the Sa-Cor T1/T0 ratio, and the bedtime Sa-Mel, the salivary hormone levels were compared between the work groups (mean, SD) using the MannWhitney *U*-test. All analyses were performed using a standard statistical programme (SPSS 15.01 for Windows, Chicago, Illinois, USA). The *P* values <0.05 were considered significant.

## 3. Results

Subjects in the ISW group were younger (39.2 years, SD 10) than those in the RDW group (42.6 years, SD 10; *P* < 0.01), and the average sleeping time was significantly (*P* < 0.001) shorter in the ISW group (5.9 hours, SD 1.0) than in the RDW group (6.6 hours, SD 0.7). Otherwise, the groups were similar. The demographic data are shown in Table 1.

The mean Sa-Cor levels at bedtime were 2.3 nmol/L (SD 1.8) and 2.5 nmol/L (SD 2.5) in the ISW and RDW group, respectively. This difference is not statistically significant. The bedtime melatonin levels, however, were significantly (*P* < 0.01) lower in the ISW group (19.7 pg/mL, SD 16.0) than in the RWD group (30.3 pg/mL, SD 27.2). The bedtime melatonin levels did not correlate to the sleeping time, Sa-Cor T1/T0 ratio in Sa-Cor, or morning sleepiness.

**Figure 1:**
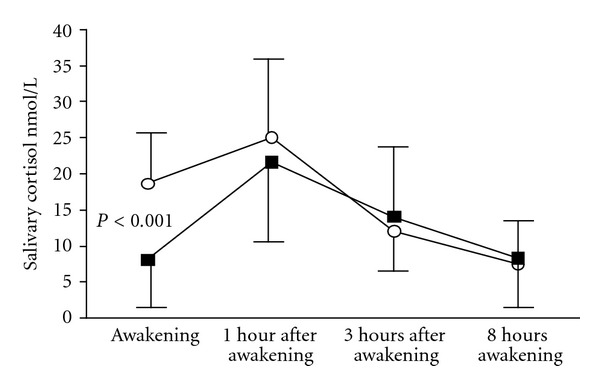
Salivary cortisol profile after awakening (AW) among Finnish media workers with irregular shift work (*N* = 65, ■) and regular daytime work (*N* = 66, ∘). *P* value for awakening samples.

The mean Sa-Cor level immediately after awakening was significantly (*P* < 0.001) lower in the IRW group (8 nmol/L, SD 6.5) than in the RDW group (18.7 nmol/L, SD 8.0). Otherwise, the cortisol profiles were similar throughout the working day (NS) (Figure 1). The mean ln Sa-Cor T1/T0 ratio in the Sa-Cor (1.2, SD 0.9) was significantly (*P* < 0.001) higher in the ISW group than in the RDW group (0.3, SD 0.5). The multiple linear regression model (Table 2) explained 32% of the variation in Sa-Cor T1/T0 ratio. Irregular shift work (*P* < 0.001), severe stress (*P* < 0.05), and shorter sleep (*P* < 0.05) were independently significantly associated with the Sa-Cor T1/T0 ratio (Table 2).

## 4. Discussion

Our study revealed clear-cut associations between the augmented Sa-Cor response during the first hour after awakening, irregular shift work, shorter sleep, and severe stress experience. In a multiple linear regression analysis, these three measures explained almost one-third of the variation of the Sa-Cor 60/0 ratio in our subjects. The levels of bedtime Sa-Cor did not differ significantly between the ISW and RDW groups. Moreover, although the bedtime Sa-Mel levels were lower in the ISW group than in the RDW group, the melatonin levels did not correlate with sleeping time, morning sleepiness, or the Sa-Cor T1/T0 cortisol ratio.

Although the awakening cortisol levels were significantly higher in the RDW group, the mean Sa-Cor levels were within published reference limits in both groups [28], and no significant differences were observed later during the day (see Figure 1).

Cortisol activation has short-term protective effects, but chronic or extreme activation may have long-term negative impacts [29]. A low basal level with high awaking responsiveness has been associated with metabolic syndrome, especially among women [30], and it is possible that shift work predisposes people to metabolic disturbances in many ways [31]. Mental stress may augment these effects.

In this study, ISW was an independent predictor of an augmented Sa-Cor T1/T0 ratio in the Sa-Cor response after awaking. No gender difference was seen in this context. Unfortunately, we did not measure Sa-Cor 30 minutes after awakening. We assume that the 30-minute values would have been higher than the 60-minute level. We also assume that the difference between the two groups would have been greater if 30-minute values had been used. We used 60-minute sampling in the morning, not 30-minute sampling, for practical reasons. More studies are thus needed to clarify the meaning of the higher Sa-Cor T1/T0 response.

We found that increased subjective stress, short sleeping time, and ISW were independent risk factors for an increased Sa-Cor T1/T0 ratio in the morning, reflecting the CAR. In a recent study, 24% of multiprofessional media personnel reported having rather much or very much stress [25]. The effects of poor recovery and stress on the CAR are controversial [4, 32, 33]. In this population of media workers, the elements of poor recovery, for example, short sleeping time, ISW, and mental stress, were correlated with increased activation of the HPA axis after waking. Irregular diurnal rhythms and shortened sleep have been found to modulate the HPA axis function [34]. An augmented CAR may predispose people to some negative health effects. Monitoring cortisol excretion may be useful among individuals who are working in occupations with a high risk of HPA axis strain.

We are not aware of previous studies measuring Sa-Cor in ISW and therefore our findings are all novel. In addition, our study has several strengths. First, subjects with the same job content were carefully randomized according to their type of working schedule. In addition, the methods used in this real-life setting were simple and can be used both in interventional studies and in follow-up studies. In occupational studies, using actigraphy may give a better estimate of the length of sleep than does a subjective estimation alone [35, 36].

Our study also has some limitations. For instance, the commonly used sample taken 30 minutes after awakening was not included in the protocol. Our first sample taken after the awakening sample was taken 60 minutes later. For this reason, we may have missed even larger differences between the groups. In addition, we used only questions to evaluate the subjective stress level. Using more questions, we could have analysed the different factors behind the stress in more detail. A limitation is also that we analysed the duration of sleep by actigraphy but the quality of sleep was not evaluated more precisely with polysomnography.

To conclude, ongoing changes in working life are rapid and profound and it is probable that some of the negative consequences of these changes have yet to emerge. Based on the present results, a stressful work environment and irregular shift work is characterized by lower awakening cortisol levels but a greater increase in cortisol excretion after awaking. It remains to be studied whether this type of reaction correlates with negative health effects in the long run.

## Figures and Tables

**Table 1:** Characteristics of media workers with or without irregular shift work.

	IRW (*n* = 65)	RDW (*n* = 66)	*P* value*
Mean (SD):			
Age, years	39.2 (10)	43.6 (10)	<0.05
Body mass index	24.4 (3.2)	24.5 (4.7)	NS
Actual sleeping time, hours	5.9 (1)	6.6 (0.7)	<0.01
Men	47% (*n* = 31)	48% (*n* = 32)	NS
Alcohol consumption (over 10 alcohol equivalent units/week)	22%	20%	NS
Single, divorced, or widowed	38%	52%	NS
Perceived severe stress	12%	9%	NS

*Statistics: Student's *t*-test for group means and chi square test for proportions.

**Table 2:** Associations of the studied items with the ratio of salivary cortisol level 60 minutes (T1) after awakening to salivary cortisol level immediately (T0) after awakening (T1/T0 ratio).

*n* = 131	*β*	SE	*P* value
Severe stress	0.48	0.21	<0.05
Shift work	0.73	0.13	<0.001
Actual sleep time	−0.15	0.07	<0.05
Gender (female)	0.20	0.12	NS
Age	−0.01	0.01	NS

Multiple linear regression. Adjusted *R* ^2^ = 0.321.
